# Impact of an educational intervention model on pesticide practices among farmers: a randomized controlled trial protocol

**DOI:** 10.1186/s12889-026-26501-6

**Published:** 2026-02-07

**Authors:** M U Tejashree, Rajalakshmi Rajendran, Shravya Chitrapady, Kapu Haritha, Sohil Khan, Sreedharan Nair, Girish Thunga

**Affiliations:** 1 Department of Pharmacy Practice, Manipal College of Pharmaceutical Sciences, Manipal Academy of Higher Education, Manipal, India; 2 Pharmacotherapeutics and Evidence Based Practice, School of Pharmacy and Medical Sciences, Griffith University, Gold Coast Campus, Queensland, 4222 Australia

**Keywords:** Farmers, Documentary, Need-based education, Pesticides, Randomized controlled trial

## Abstract

**Background:**

Pesticide poisoning and unsafe handling among pesticide users, particularly with farmers, pose a significant threat to both public health and the environment. Despite efforts to improve farmers Knowledge, Attitude and Practices, most interventions lack tailored education and sustained follow-up, limiting their effectiveness. This trial assesses the impact of need-based educational intervention with educational documentary, improving safe pesticide practices among farmers in Karnataka, India.

**Methods:**

The study is a multicentric randomized controlled trial, which will be conducted across 4 phases in Udupi, Mysuru, and Shivamogga districts of Karnataka. Phase 1 features a cross-sectional study design of 175 farmers to evaluate their baseline knowledge, attitudes, and practices on usage of pesticides. Phase 2 employs semi-structured interviews with 20–30 farmers and 10–20 key stakeholders, including pesticide dealers, scientists from Farm Science Centers [Krishi Vigyan Kendra (KVK)], and joint directors of agriculture to explore pesticide handling approaches, challenges, and community-specific needs while handling pesticides. The findings from Phases 1 and 2 will help in development and validation of an intervention in the form of educational video in Phase 3 which will be piloted. Phase 4 involves implementing the validated intervention and conducting long-term follow-up to assess the impact and sustainability. Phase 4 will be a two-arm, single-blind, parallel-group randomized controlled trial, with villages randomized 1:1 into intervention and control groups. The intervention group will receive a 15–20-minute educational documentary in the local language, while the control group will receive no intervention. The outcomes will be evaluated based on changes in Knowledge, Attitudes, and Practices (KAP) scores, with assessments conducted immediately post intervention, at 6 months, and at 12 months from the date of intervention. These will be correlated with outcomes including quality of life and laboratory parameters.

**Discussion:**

The study aims to provide robust evidence on the role of need-based educational intervention in promoting safe pesticide practices among farmers in rural region. The primary aim of the study is to develop practical strategies to enhance public health and farmer safety by addressing gaps and contextual relevance. It seeks to improve farmers’ knowledge, attitudes, and practices regarding pesticide use, ultimately enhancing their quality of life and promoting better health and safety outcomes.

**Trial registration:**

The trial is registered at Clinical trials Registry India with the trial registration number CTRI/2024/06/069678.

**Supplementary Information:**

The online version contains supplementary material available at 10.1186/s12889-026-26501-6.

## Background

India ranks among the largest pesticide users globally, with pesticide-related disorders emerging as a significant public health concern [1]. The adoption of intensive agricultural practices and reliance on synthetic pesticides has led to extensive occupational exposure, increasing the risk of severe health outcomes, including neurological disorders [2].

Around 4.19 million metric tons of pesticides were used worldwide in 2019, with China leading the consumption at approximately 1.76 million metric tons. In Southeast Asia, pesticide usage continues to rise annually, with 20% of developing countries—including Cambodia, Laos, and Vietnam—identified as major consumers [3]. India stands as a leading pesticide producer in Asia, manufacturing around 90,000 tons annually and holds 12th position in the world for manufacturing of pesticides [1].

Between 2010 and 2014, the global average cost-benefit ratio was 0.645 g of total pesticides per kilogram of crop yield. The average annual pesticide consumption during this period was 2.784 kg ha–1 worldwide, while India reported a significantly lower average of 0.26 kg ha–1 [4]. 

The WHO classifies highly toxic pesticides into categories such as Class I and Class II, including organophosphates and carbamates, which have been linked to significant health effects. These include health risks on central nervous system (CNS), skin disorders, cancer, and respiratory problems [5]. A recent systematic review with meta-analysis from 2022 [6], examined the association between pesticide exposure and allergic diseases—such as asthma, allergic rhinitis, and atopic dermatitis in children and adolescents. The findings indicated that children and adolescents exposed to pesticides had a two-fold increased risk of asthma symptoms [OR = 2.14; 95% CI: 1.26–3.64] [6].

South Asia has one of the highest pesticide usage rates globally, driven by its agricultural demands and variations in climatic conditions. Although alternatives to chemical pesticides are available, their limited efficacy has led to a greater dependence on synthetic insecticides. While these products play a crucial role in enhancing agricultural productivity, their improper and excessive use has become increasingly common, contributing to persistent health problems among farmers [7, 8].

Additionally, pesticide residues can enter households through various pathways, including contaminated clothing, ingestion, inhalation, dermal absorption, floor dust, and other take-home routes [9, 10]. A 2023 study conducted in Indonesia among a population of 105 farmers reported acute health issues associated with pesticide exposure, with 41.9% experiencing Occupational Skin Diseases [OSD] [11, 12] and 28.78% suffering from dry-eye syndrome [11, 13]. Furthermore, a 2024 meta-analysis of 38 studies involving over 118,000 children revealed a 24% increased risk of asthma and a 34% higher risk of wheezing among children exposed to pesticides [14, 15].

Long-term exposure has been frequently associated with chronic neurological disorders, including Alzheimer’s disease [16, 17], Parkinson’s disease [18, 19], cognitive impairments [3.4%], and neuropathies [61.7%] [11]. Additional neurological problems include seizures, epilepsy, and cancer [20, 21]. Children are particularly susceptible to pesticide exposure, which is linked to growth retardation, behavioural disorders, allergies, and other adverse health effects [19, 22].

Karnataka, a key agricultural state in India, encompassing a geographical area of 190,500 km² [19.05 million hectares], with 65% of its population relying on agriculture as their primary livelihood [23]. Pesticide application is a widespread practice among farmers in this state. According to the 2022–2023 annual report from the Agriculture Department of Karnataka, overall pesticide usage has slightly declined over the past decade. Nevertheless, Karnataka remains among the states with the highest per-hectare expenditure on pesticides. This reflects the intensive application of these chemicals on major crops such as paddy, ragi, maize, sorghum, red gram, Bengal gram, sunflower, groundnut, cotton, and sugarcane [24].

Karnataka state is home to 8.68 million farming families, cultivating 118,050 km² (11.81 million hectares) of rainfed and irrigated land [25]. The subtropical climate of Karnataka, which is prone to insect breeding, further increases the demand for pesticides. Despite this, the adoption rate of chemical pest control methods in Karnataka is relatively low [22%] compared to other agricultural states, highlighting a potential gap in awareness or accessibility to modern educational interventions regarding pesticide usage and its implications [26].

The increasing reliance on pesticides has led to unsafe handling, improper mixing, inadequate spraying techniques, insufficient use of personal protective equipment (PPE), and a lack of expertise, all of which pose significant health risks [27]. Although the dangers of pesticide poisoning have been addressed through various educational interventions, including seminars, pamphlets, puppet shows and lectures, the effectiveness of these efforts is often compromised by low literacy rates, limited farmer engagement, and insufficient follow-up [28].

Based on the findings of the 2021 systematic review [29], educational interventions were less successful in changing participants behaviours and lowering exposure risks, despite successfully improvement in their knowledge and attitudes. This was observed primarily because of the limited follow-ups, that lasted less than one month or six months. Furthermore, none of the studies explored the use of technological interventions [29]. The long-term sustainability among the reported study is still unclear, as evidenced by the fact that only 7 of the 21 studies [29], that were part of the evaluation concentrated on knowledge and beliefs and had follow-up periods longer than six months [29, 30].

Besides, studies utilizing need-based educational approaches are scarce, and when such studies are conducted, they frequently lack adequate follow-up, thereby limiting their effectiveness [31]. Regardless of the study design or type of intervention implemented, research consistently suggests that the effectiveness of educational programs in agricultural settings is often hindered by insufficient follow-up and a lack of context-specific customization. These limitations undermine the long-term impact and sustainability of the interventions, emphasizing the need for comprehensive strategies that include ongoing support and monitoring [29, 32].

The proposed multicentric randomized controlled trial (RCT) aims to address the challenges faced by farmers by implementing a mixed-methods approach. This study incorporates a video-based educational intervention with periodic long-term follow-ups, which enhances both the effectiveness and the sustainability of the knowledge gained by farmers. The need-based educational strategy ensures that the intervention targets specific knowledge gaps and practices, thereby improving the effectiveness and long-term adoption of safe pesticide practices.

It is hypothesized that by focusing on KAP and integrating qualitative insights from relevant stakeholders, alongside visual educational tools, the study will offer a sustainable and contextually relevant solution to the persistent issue of pesticide poisoning and its associated health impacts.

## Objectives

The objectives of this study include:

1. To assess the KAP among farmers and correlate it with respect to health-related Quality of Life (QoL) along with biochemical parameters.
2. To conduct qualitative interview among farmers and respective stakeholders to develop need-based education intervention.
3. To assess the impact of educational intervention on KAP and quality of life through multicentric RCT.

### Trial design

This study adopts a multi-phase, mixed-methods approach to address pesticide safety practices among farmers in Karnataka state. The study will be conducted in 4 different phases. Phase 1 is an analytical cross-sectional study, designed to evaluate the baseline knowledge, attitudes, and practices (KAP) of farmers using structured questionnaires. Phase 2 involves qualitative face-to-face interviews to gain deeper insights into the behavioural and contextual factors influencing pesticide usage and safety practices.

Phase 3 involves development and validation of need-based education module, where selection of experts will be carried out by purposive sampling technique. Phase 4 is a multicentric RCT, where 3 districts that includes 6 villages will be selected based on randomization. This study will be conducted as a two-arm, single-blind, parallel group with simple randomization of the districts and cluster randomization of farmers. The allocation ratio is 1:1, with one arm receiving the educational intervention and the other serving as a control group with no intervention. The outcomes will be assessed in terms of mean difference with the pre and post assessment in terms of KAP. The trial adopts a superiority framework [33] to assess the effectiveness of the educational intervention in improving pesticide safety knowledge, attitudes, and practices among farmers.

**Fig. 1:**
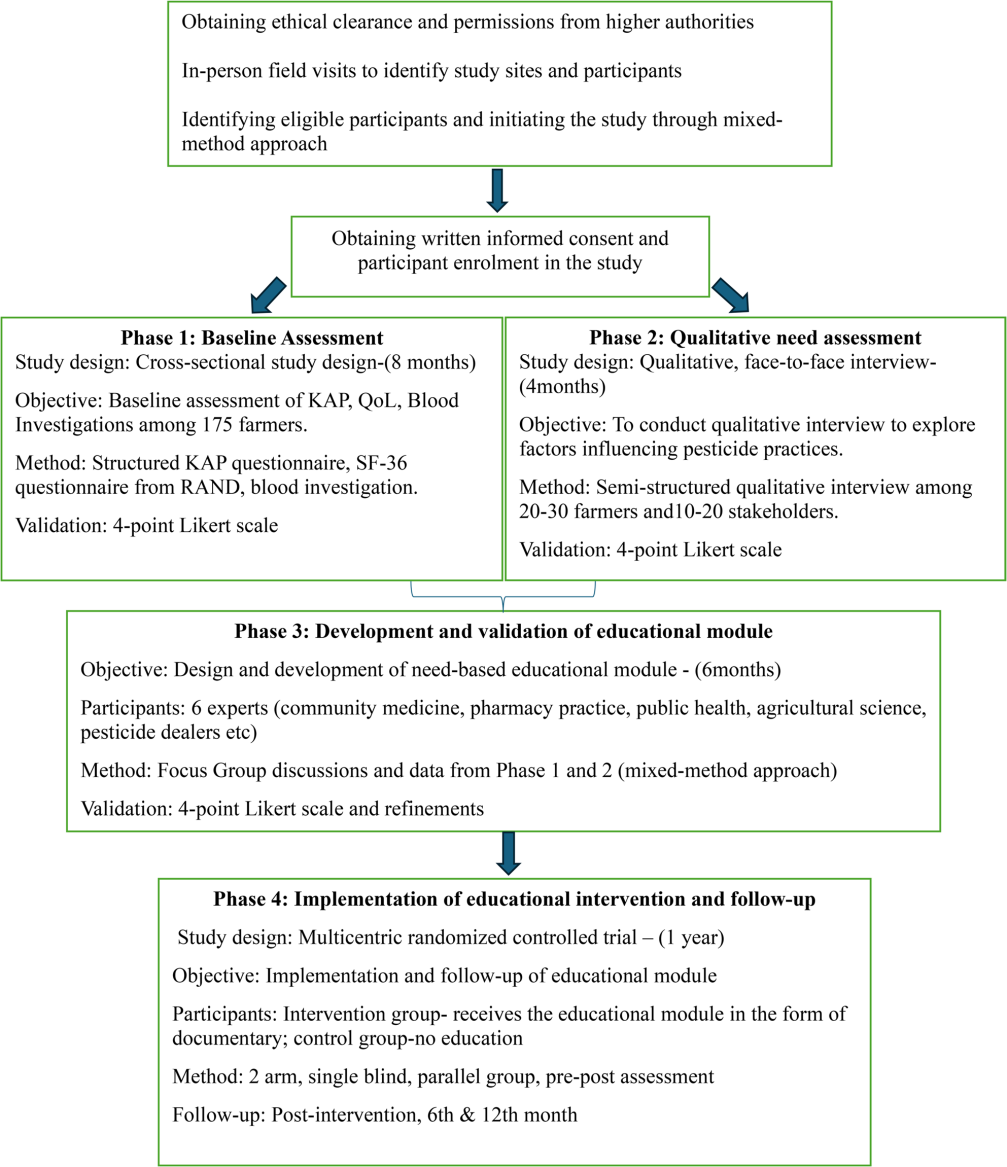
Flowchart depicting trial process from recruitment to randomisation

The below Fig. 1 depicts the trial process from recruitment to randomisation.

## Methods: Participants, interventions, and outcomes

### Patient and public involvement (PPI) – GRIPP2-SF

The study incorporates patient and public involvement (PPI) following the GRIPP2-SF (Guidance for Reporting Involvement of Patients and the Public – Short Form) [34], to ensure that the research is relevant, acceptable, and beneficial to the target population. Farmers will be involved to develop and validate the educational intervention based on their lived experiences with pesticide use. Farmers, agricultural officers, and community representatives will be engaged in the study, through focus group discussions to shape the study design, intervention content, and outcome measures. Their insights and experiences can help in the betterment of the mode of educational delivery and strategies to enhance adherence. During implementation, public representatives will be involved to support participant recruitment and provide feedback on intervention practicality. PPI members will also contribute to interpreting results, ensuring contextual relevance. Study findings will be disseminated through community meetings at village halls or community meetings. Reflections on PPI impact, including benefits and challenges, will be documented to develop future research.

### Study setting

The study will be conducted in 4 different phases. After a thorough literature review, it was evident that pesticide exposure and residue contamination were particularly high in certain regions of Karnataka. Data on pesticide exposure frequency were gathered from Farmer Producer Organizations (FPOs), which further supported the identification of high-risk areas. Additionally, factors such as crop diversification and regional agricultural practices were considered for study site selection. Based on these evidences, three districts—(a) Udupi, (b) Mysuru, and (c) Shivamogga— [31, 35, 36] were selected as the primary study sites. These districts have been reported to experience significant pesticide use, improper handling practices, and contamination risks in food and water systems. Prior studies have highlighted their vulnerability to pesticide-related health hazards, justifying their selection for this study. Furthermore, importance was given to interior villages within these districts to minimize contamination from external factors and focus on the specific challenges faced by the target group [31, 35, 36].

As a result, three districts that comprise various taluks were identified. Importance was given to interior villages in these taluks in order to minimize contamination and keep the study’s focus on the particular obstacles faced by the target group.

In-person field visits for data collection and laboratory investigations in these regions will be carried out. Additionally, permissions and ethical clearances from the appropriate authorities have already been obtained. In accordance with ethical guidelines, all participants will get a clear Participant Information Sheet (PIS) and Informed Consent (IC) form before enrolling to the study. The study complies with WHO and CONSORT 2025 guidelines and is registered with the Clinical Trials Registry India [CTRI] [37] with the registration number CTRI/2024/06/069678. The trial was registered on 28/06/2024 with the reference url https://ctri.nic.in/Clinicaltrials/regtrial.php? modid=1&compid=19&EncHid=55173.32324.

**Fig. 2:**
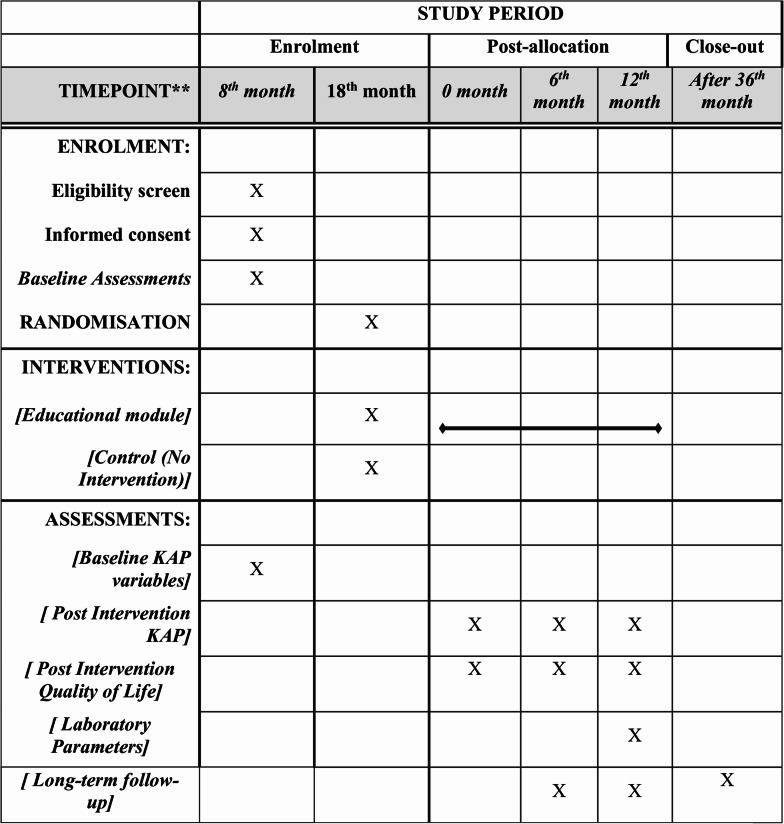
SPIRIT Checklist 2025 for Timeline and Assessment Schedule of a Multicentric Randomized Controlled Trial on Impact of an educational intervention model on pesticide practices among farmers: A randomized controlled trial protocol in Karnataka, India

The study follows the SPIRIT 2025 guidelines [38] to ensure transparency and standardization. Details are provided in the supplementary file 1. Additionally, The SPIRIT 2025 figure format has been presented in Fig. 2 to illustrate the participant timeline and study procedures.

### Eligibility criteria

Inclusion criteria for study participants comprise: (a) Farmers who are actively engaged in regular pesticide spraying; (b) Farmers who are willing to provide informed consent (c) Farmers with a minimum of 5 years of experience in agriculture and pesticide spraying; (d) Farmers aged between 18 and 80 years, irrespective of their gender. Following this, any farmers who unwilling to provide consent and who are just involved in agriculture but lack pesticide spraying will be excluded from the study.

### Intervention

The primary component of the intervention will be a 15–20-minutes educational documentary in the local language. Topics like safe pesticide usage, proper storage, disposal methods, and associated health risks will be covered in this documentary. These elements will be presented through a story that will be displayed as a short documentary or movie among the farmers. Cluster sampling method will be utilised for selecting participants based on characteristics including age, education, and years of experience with pesticides or agriculture. The intervention will be provided in the intended villages at community centres or village halls.

The control group will not receive any educational intervention during the study period. Participation records will be maintained to track adherence to intervention, and any participant’s decision to withdraw from the study will be documented. If withdrawn, data collected up to the point of withdrawal will be retained for analysis. The appropriate reason will be documented for withdrawal if available.

To maintain the integrity of the study, participants will be requested to refrain from engaging in other pesticide-related educational initiatives, although they may continue their regular farming activities. Adherence to the intervention and pesticide practices will be monitored through regular follow-up calls, personal visits, and collaboration with field officers, community leaders, and local organizations.

The detailed participant timeline has been provided in Fig. 2, following the SPIRIT 2025 format [39] .

### Outcomes

The outcomes for this trial will be evaluated through quantitative and qualitative measures.

### Primary outcomes

The primary outcomes of the trial are (i) Knowledge, Attitude, and Practice (KAP) scores, assessed using a validated KAP questionnaire, and (ii) Quality of Life (QoL) scores, evaluated using the SF-36 Questionnaire. Both outcomes will be measured at baseline, post-intervention, and periodic follow-ups, with results analysed as mean changes over time and correlated with laboratory findings to provide a comprehensive understanding of the intervention’s impact.

### Secondary outcomes

The secondary outcomes of this trial include (i) adherence to safe pesticide handling practices, (ii) knowledge on first aid for unintentional or occupational pesticide exposure. Additional outcomes comprise (iii) frequency of direct pesticide exposure, assessed through self-reported practices and associated health hazards during follow-up, and (iv) development and dissemination of a localized educational tool, evaluated qualitatively, to promote sustainable knowledge and practices. These outcomes have been selected for their clinical relevance in addressing the short- and long-term health risks associated with indiscriminate pesticide usage, enhancing overall safety, and fostering effective behavioural change among farmers. These outcomes will be assessed by an investigator through observation of the practices and using the self-reported checklists.

### Participant timeline

The trial will be conducted over a period of 3 years in 4 phases. During the phase 1, with duration of 8 months, 175 farmers will be recruited to assess their baseline knowledge, attitudes, and practices (KAP) regarding pesticide usage, which will be correlated with quality-of-life and biochemical parameters.

In the phase 2, which will last 4 months, around 20 to 30 farmers will participate in qualitative interviews to share their needs and perspectives on pesticide usage patterns, disposal methods, and storage practices. Additionally, 10 to 20 key stakeholders, including pesticide dealers, scientists from Farm Science Centre [Krishi Vigyan Kendra’s (KVK’s)], joint directors of agriculture, assistant director of agriculture, subject matter specialist [SMS] will be involved from the selected villages across different districts, to gather qualitative insights and recommendations regarding pesticide usage patterns.

Following this, phase 3 will be conducted over 6 months, which focuses on the development and validation of the educational module. This phase will be followed by a pilot implementation lasting further 6 months.

The final phase 4 will be executed as a randomized controlled trial (RCT), where the validated educational module will be implemented in the selected villages for a specific duration of 2 months. Participants will be followed up at intervals of 6 months and 12 months. The total duration of phase 4 will be 1 year.

### Sample size

The sample size for each phase of the study was calculated distinctly, tailored to the study design and objectives. A formal power calculation was conducted to ensure appropriate statistical power for detecting meaningful differences.

For phase 1, the sample size was determined using data from Sam KG et al. (2007) [31], a study conducted in Udupi, Karnataka, India, which reported a baseline Knowledge, Attitude, and Practice (KAP) score of 30.88 with a standard deviation of 10.33. The required sample size was calculated using the following formula for comparing two means:

$\mathrm{n}\;= \;2\times\;[(\mathrm{Z}_{\alpha/2}\;+\;\mathrm{Z}_{\beta})\;/\;\mathrm{Z}_{\delta}\;]^{2}$ where, n = required sample size per group, Z_α/2_ **=** standard normal deviate for a given significance level (e.g., 1.96 for 5% significance level), Z_β_ = standard normal deviate for the desired power (e.g., 0.84 for 80% power), σ = standard deviation of the outcome variable (10.33 from prior study) and Z_δ_ = expected mean difference in the effect size.

A statistical power of 80% was chosen, which is widely considered an acceptable standard in health and social science research [40]. A power of 80% indicates that there is an 80% probability of detecting a true effect if one exists, while maintaining a Type I error (α) of 5%. Accounting for a 10% dropout rate, a total of 175 farmers will be selected using the stratified sampling method for the KAP assessment, quality of life evaluation, and laboratory investigations.

In phase 2, with approximately 20-30 farmers and 10–20 stakeholders from various villages across the selected 3 districts will be involved in the qualitative interviews for the need assessment phase.

Phase 3 focuses on the development and validation of the educational intervention by 6 experts from various domains including experts from agricultural sciences, pharmacy practice, community medicine, public health, and emergency medicine.

For Phase 4, the sample size was determined based on findings from a pilot study involving 12 farmers, with equal allocation to intervention and control groups in a simple randomized controlled trial (RCT) design. The final sample size was calculated using the power calculation formula for a paired t-test:$\mathrm{n}\;=\;2\times\;[(\mathrm{Z}_{\alpha/2}\;+\;\mathrm{Z}_{\beta})^{2}\;*\;2\sigma^{2}\;/\;\mathrm{Z}_{\delta}\:^{2}]$, where, n = required sample size per group, Z_α/2_**=** standard normal deviate for a given significance level (e.g., 1.96 for 5% significance level), Z_β_ = standard normal deviate for the desired power (e.g., 0.84 for 80% power), σ = standard deviation of the outcome variable (10.33 from prior study) and Z_δ_ = expected mean difference in the effect size.

To ensure the effective implementation of the educational intervention, farmers will be stratified by age and education level.

### Recruitment of participants

The recruitment process will follow distinct approaches for each phase.

In phase 1, farmers will be recruited through purposive sampling method, utilizing suggestions from Farmer Producer Organizations (FPOs) and recommendations from District Agricultural Officers. Participants will be briefed on the study objectives, and informed consent will be obtained. Each participant will be provided with a Participant Information Sheet (PIS), which outlines the study’s purpose, procedures, potential risks, and benefits. Recruitment will proceed only after obtaining Institutional Ethics Committee (IEC) approval. Samples will be collected from 3 districts, with an equal number of participants from each district. The districts will be selected based on pesticide usage, crop types, pesticide poisoning exposure (intentional and accidental), farmers’ experience, education, and age. Eligible participants will be enrolled after meeting the inclusion criteria, completing baseline questionnaires, and undergoing laboratory investigations.

Phase 2 will involve qualitative interviews to assess farmers’ perceptions and needs, with consent obtained for audio recording of 20–30-minute sessions. Farmers from Phase 1, with substantial experience in pesticide spraying, knowledge of pesticide poisoning, and adequate educational qualifications, will be selected from the three districts to participate in the interviews. Stakeholders will include FPO heads, agricultural officers, and other community leaders involved directly or indirectly with the farmers in the pesticide handling and crop management.

In phase 3, for recruitment of the experts for the validation of the need-based educational module, experts will be selected by purposive sampling method based on their area of expertise. This includes experts from community medicine, public health, sociology, audio-visual section, agricultural officer and farmer representative. A focus group discussion will be carried out during the recruitment and interviews will be done to understand the needs of farmers based on opinions from experts. And finally, data from phase 1 and 2 along with expertise will be gathered to develop a need-based educational intervention.

In phase 4, the same villages from Phase 1 will be selected for recruitment. The districts will be randomized into control and intervention groups. Farmers will be identified using cluster sampling, with stratification based on age and agricultural experience. The intervention will be delivered to the experimental group at village halls or community centres. The cluster sampling approach ensures that participants are diverse in terms of age, educational level, and years of experience in agriculture allowing for a balanced representation across the groups.

## Methods: assignment of interventions (for controlled trials)

### Random sequence generation and randomization

The allocation sequence will be generated using a computer-generated random number table through simple randomization. Participants who enroll in the study will be assigned with a unique identification code in alphabetical order. In Phase 4, villages will first be randomized into intervention or control groups. Within each group, participants will be categorized using a cluster randomization technique, accounting for confounding factors such as age, gender, education level, and agricultural experience. Stratification will further ensure balance by considering key variables like crop varieties and pesticide spraying techniques.

### Blinding

This is a single-blinded, double-arm, parallel-group study design, where only the participants will be blinded to their group assignment (intervention or control). This approach minimizes the risk of performance bias among participants. Outcome assessors, data analysts, and other study personnels will not be blinded as it is an educational interventional study.

## Methods: data collection, management, and analysis

### Data collection and management

In phase 1, data will be collected in person through face-to-face interactions using baseline questionnaires, including the KAP questionnaire and the SF-36 Quality of Life (QoL) questionnaire from RAND Health. Additionally, qualitative interviews will be conducted, and laboratory tests (blood samples) will be collected from enrolled participants. All quantitative data will be stored in OpenEpi version 3.1 software and later transferred to SPSS version 20.0 (IBM, New York, NY, USA) for statistical analysis.

In phase 2, qualitative interviews will be recorded with prior consent, transcribed, and stored securely on a password-protected device. These recordings will be transcribed and translated to verbatim, and themes and sub-themes will be developed using NVivo software for qualitative analysis.

In phase 3, after developing the educational documentary, its validation will be conducted using a Likert scale (ranging from 1 to 4) and face-to-face focus group discussions (FGDs). These discussions will be recorded, transcribed, translated, and analysed to extract themes and suggestions, which will be incorporated to refine the documentary.

In phase 4, pre- and post-intervention questionnaires will be administered to both the intervention and control groups, mirroring the data collection process from Phase 1. The collected data will be stored in OpenEpi version 3.1 software and subsequently analysed using SPSS version 20.0 (IBM, New York, NY, USA).

Throughout the study, participant identities will remain anonymized, and all data will be coded for confidentiality. At the conclusion of the study, data will be discarded as per study protocols. Missing data, such as those resulting from loss to follow-up or participant withdrawal, will be documented with appropriate justifications and reported in the results.

### Statistical analysis

All data collected through the KAP and QoL questionnaires will be entered into SPSS version 20.0 (IBM, New York, NY, USA) for statistical analysis. Paired t-tests will be performed for continuous variables to compare pre- and post-intervention results. For improvements in quality of life and changes in laboratory parameters, the Wilcoxon Signed Ranks Test will be used. The results from the KAP surveys will be presented as mean ± standard deviation (SD). Qualitative interview data will be analysed using NVivo software, with thematic analysis to identify key patterns, insights and to understand the needs and perception of the farmers. Subgroup analyses based on confounding factors such as age, gender, and education level will be conducted to assess differential effects of the intervention. An intention-to-treat analysis will be performed and per-protocol analysis for participants deviating from the protocol.

## Discussion

Pesticide poisoning is a severe health condition affecting people worldwide. Different possibilities for pesticide safety and awareness have become available through interventions in recent years. Nevertheless, only few of these applications have been used in real-world settings. The widespread and ongoing use of these pesticides presents major safety risks to both the end user who consumes the product and those who handle them. While many educational programs have been developed to address these issues, yet studies have demonstrated that their effectiveness in enhancing KAP, safe practices are not optimized. The barriers are numerous, including socioeconomic obstacles, behavioural resistance, and limited access to resources, all of which demand further for customized approach to enhance compliance.

Furthermore, a systematic emphasis on establishing knowledge retention and developing it into long-lasting routines has frequently been lacking within education interventions. Evidences suggests awareness levels have increased, but retention of information remains a challenge requiring long-term follow up studies [41]. Methodological delivery of educational intervention plays a crucial role in retention of information. Due to various agricultural engagement, farmers are unlikely to have sufficient time to attend meetings, workshops, trainings, or lessons regarding the safe usage of pesticides. Thus, documentaries are one of the best-regarded visual learning tools, since it allows individuals to watch them whenever they’ve got leisure time, discuss the contents with others, and view them multiple times as suits them [42–44]. Hence, the study focusses not only on what should be included, but also on the kind of manner the target population like. However, through an RCT design, the opinions and perceptions of key pesticide sprayers will be included using a mixed-method approach to enhance pesticide information retention. With this, the design and findings from this trial will have an external applicability in relation to pesticide education in rural settings.

## Conclusion

The study aims to educate farmers through a need-based approach, where data will be collected directly from farmers to identify specific inducer needs. This initiative is designed not only to enhance their knowledge but also to improve their practices in minimizing hazards associated with pesticide usage. A key focus of the study design is to assess knowledge retention and behavioural changes over time, ensuring the long-term sustainability. To our best knowledge, this is the first study from South India region to evaluate the implementation of a need-based educational intervention using a mixed-method approach within an RCT design.

## Supplementary Information

Supplementary Material 1.

## Data Availability

Not applicable, as this is a protocol manuscript.
